# Comparative Efficacy of Commercial Antilice Shampoos Against Head Lice in a High-Prevalence Primary School in Thailand

**DOI:** 10.1155/japr/2770901

**Published:** 2025-03-28

**Authors:** Sirima Kitvatanachai, Utsanee Supcharoengoon, Nutnicha Suphakhonchuwong, Aree Taylor, Pochong Rhongbutsri

**Affiliations:** ^1^Department of Hematology, Clinical Microscopy and Parasitology, Faculty of Medical Technology, Rangsit University, Pathum Thani, Thailand; ^2^Department of Preclinical Science, Faculty of Medicine, Thammasat University, Pathum Thani, Thailand

**Keywords:** antilice shampoo, efficacy of treatment, head lice, primary schoolgirls

## Abstract

This is a cross-sectional study that is aimed at evaluating the efficacy of antilice shampoo against head lice infestation in primary schoolgirls at level 3–6 (aged between 9 and 12 years) with a high prevalence of infestation in Pathum Thani Province and which also reported a current prevalence of lice infestation in this school. The study was conducted during January–March 2023. A total of 356 schoolchildren were screened for lice infestation. The prevalence rate of lice infestation was found in 78 students (21.9%) from all schoolchildren. Girls (50.3%) showed a higher rate of lice infestation than boys (0.5%) with a significant difference (*p* < 0.05). Most lice-infested girls (100%) reported itching. Seventy-two of 77 infested girls (93.5%) agreed to use shampoos for lice treatment. Two types of commercial shampoos were considered for lice treatment: (1) chemical (permethrin 0.5% W/V) antilice shampoo and (2) herbal antilice shampoo, since it is cheap and available in the market. The results of chemical (permethrin 0.5% W/V) antilice shampoo showed similar efficacy for lice treatment as herbal antilice shampoo with no significant difference in statistics (*p* > 0.05). Using the antilice shampoos consecutively 3 times demonstrated the highest efficacy of treatment (73.0%), followed by 2 times (34.3%), and the lowest efficacy of treatment was a single application (5.6%). No serious side effects from both shampoos in participants: 2 cases of itching and 1 case of the burning sensation. This indicated that in the area of the high prevalence of lice infestation, herbal antilice shampoo might be an alternative choice for lice treatment. However, we recommended using it once a week 3 times consecutively to reach the highest efficacy for treatment. In addition, this treatment should be extended to family members with lice infestation.

## 1. Introduction

Pediculosis still remains a worldwide public health problem, ranging from 0% to 78.6% in different countries, social or economic status [1–4]. Direct contact with an infested person and shared personal items such as hairbrushes, combs, clothing, hats, or towels might be the way to spread head lice [4]. Common clinical symptoms are pruritus, popular urticaria, and itching [5]. Heavy and chronic infestations lead to anaemia [6]. It also causes psychological stress in children and parents [7]. Head lice is commonly found in school-aged children between 3 and 12 years old [1, 4, 8, 9] and is especially present in girls more than boys [10–12].

The prevalence of head lice infestation in Thailand varies from 15.1% to 86.1% [13, 14]. Currently, a high prevalence of 68.7% was found in schoolgirls in a suburban school in Pathum Thani, in the central part of Thailand, which was unable to solve this problem for several years. No significant potential factors were found associated with head lice infection in this area [12].

Various ways to eliminate head lice include using a fine comb and cutting pupils' hair [5]. Pediculicides based on neurotoxic insecticides such as malathion, carbaryl, and permethrin are widely used for head lice treatment. However, head lice resistance to insecticides has been reported worldwide [15–18]. The standard for pediculosis treatment in Thailand uses three drugs which are recorded in a list in the National List of Essential Medicine: (1) benzyl benzoate lotion, (2) permethrin, and (3) ivermectin [19]. The adverse effects of chemical pediculicides, such as irritation and burning sensation on the scalp, have been noted [20, 21], and also, 25% benzyl benzoate lotion is cumbersome for treatment of head lice because of the need to apply it overnight for treatment. Treatment failure can be caused by louse resistance to insecticides, incomplete exposure time, or insufficient dosage [22–24].

Natural ingredients or herbal antilice products have milder side effects and less toxicity [25]. Many medical plants showed a high efficacy for the treatment of lice including *Acorus calamus* Linn., *Phyllanthus emblica* Linn., and *Zanthoxylum limonella* Alston [25]; Annona squamosa seeds [26]; and Indian mulberry leaf mixed with Galanga [27]. In contrast, Stemona root crude extract or mixed plant extracts showed lower efficacy (< 50% mortality) in killing head lice than 4% dimeticone (kill 100% head lice in 15 min) [28].

Previously, a single application of 20 mL chemical antilice shampoo (permethrin 0.5% W/V) was provided to schoolgirls with lice infestation once a year at Lak Hok Subdistrict, Pathum Thani. However, the prevalence of infestation remained high; one reason might be due to improper treatment, according to Tanthanapanyakorn et al. Two thousand and nineteen recommends use over a longer period, resulting in better efficacy of pediculosis elimination [29].

It is worth finding alternative treatments such as commercial herbal antilice shampoos, which (1) contain a potential ingredient against head lice, (2) can be applied more often, (3) are more economically viable and offer greater safety than commercial chemical shampoos, (4) take a shorter time to apply on the scalp (5–10 min) than standard drugs for lice treatment, and (5) can be reapplied several times as routine shampoo use. This study compared the efficacy of herbal and chemical antilice shampoos in primary school girls in Pathum Thani Province.

In addition, find the most effective number of applications of antilice shampoo to eliminate head lice.

## 2. Materials and Methods

### 2.1. Ethics Statement

The study design and protocols were approved by the Human Ethical Review Committee of Rangsit University, Thailand (ethical clearance no. RSUERB 2022-119).

Before participation in shampoo treatment, signed, written, and informed consent forms were provided by parents/guardians of the schoolchildren. At the end of this study, children who were untreatable with shampoos were informed to see a doctor for proper treatment.

### 2.2. Study Design and Lice Infestation Screening

A cross-sectional study was performed during January–March 2023. The screening of the lice infestation cases was conducted in the primary schoolchildren Levels 3–6 (aged between 9 and 12 years) in a suburban school in Pathum Thani Province, which previously reported a high prevalence of head lice. The results of lice infestation were recorded.

The active lice infestation cases were investigated by using a fine-tooth comb based on visual detection of the living adult or nymphal stages or viable nits under natural sunlight [30–32]. A positive infestation was recorded as at least one active infestation [31]. The result was recorded as negative for lice infestation if the nymphs or adults or viable nits were not found [33].

### 2.3. Study Participants for Comparative Efficacy of Commercial Antilice Shampoos Against Head Lice Infestation

All infested girls were asked to participate by using antilice shampoos and obtained an agreement from parents/guardians. Girls who were absent from school on the day of screening, did not agree to use shampoos, or had an allergic reaction to the shampoos were excluded from the study.

### 2.4. Comparative Efficacy of Commercial Antilice Shampoos

The eligible infested girls were randomized into two groups of equal size by using odd and even no. of infested cases at each school level. Two economically viable and commercially available antilice shampoos were chosen: one chemical shampoo with permethrin 0.5% W/V ingredient and another antilice herbal shampoo (sugar apple, tuba root, and Siamese neem tree mixture) which are both available locally and at low cost. Seventy-two infested girls were randomized 1:1 to receive either permethrin shampoo or herbal antilice herbal shampoo. Thirty milliliters of shampoo was provided to all participants with instructions on how to use the shampoo. The instruction was to briefly apply the shampoo on the scalp, leave it for 5 min, rinse with water, air dry, and then comb the hair with a fine comb to remove the dead lice. The number of negative results was recorded after treatment for 7, 14, and 21 days to assess the efficacy of antilice shampoo. If any lice infestation still showed a positive result, the same antilice shampoo was applied for the second and third treatments. After the 3rd treatment, if the results remained positive, the students were suggested to see the doctor for the proper treatment.

### 2.5. The Side Effects and Satisfaction of Using Antilice Shampoo

The side effects of antilice shampoo, including dryness, redness, and irritation, are measured. These side effects were recorded after the application of treatment. At the end of treatment, satisfaction with the antilice shampoo was evaluated by personally asking questions with three levels of satisfaction: (1) like, (2) undecided, and (3) dislike.

### 2.6. Reinfestation of Head-Lice in 2 and 12 Months

To investigate the reinfestation of lice, the treated girls were re-examined after successful treatment, in 2 and 12 months. The number of reinfested cases was recorded.

### 2.7. Data Analysis

Data were analyzed using the IBM SPSS software for Windows (Version 21.0). Data were expressed in descriptive statistics and used to describe the prevalence of lice infestation, cure rate, and side effects in percentage.

 $$\begin{array}{c} \text{The prevalence of lice infestation }\left(\%\right)=\frac{\text{Number of positive girls}}{\text{Total no}.\text{of samples}}\times 100 \end{array}$$

The efficacy of antilice shampoo was evaluated by the absence of lice and viable nits at Days 7, 14, and 21 after treatment.

 $$\begin{array}{c} \text{The efficacy rate of shampoo }\left(\%\right)=\frac{\text{Number of infested girls cured after treatment}}{\text{A total no}.\text{of infested girls included in treatment}}\times 100 \end{array}$$

To observe the reinfestation rate after 2 and 12 months,

 $$\begin{array}{c} \text{The reinfestation rate}\left(\%\right)=\frac{\text{Number of re}-\text{infested girls after treatment in }2\text{ and }12\text{ months}}{\text{Number of infested girls cured after treatment}}\times 100 \end{array}$$

To compare the efficacy between two antilice shampoos, the chi-square test was used and considered significant at a *p* value ≤ 0.05.

## 3. Results

### 3.1. Head Lice Infestation in a High Prevalence Primary School Levels 3–6 in Pathum Thani Province

Three hundred fifty-six out of 400 students (89.0%) from primary school at Levels 3–6 in a suburban area of Pathum Thani were screened for head lice infestation. The prevalence of head lice infestation was found in 78 out of 356 students (21.9%). Seventy-seven of 153 girls (50.3%) showed a higher head lice infestation than boys (1 out of 203 (0.5%)) with statistical significance (*p* < 0.05).

The distribution of head lice infestation in primary school Grades 3–6 (the age range between 9 and 12 years) was not significantly different in each level (*p* > 0.05). The history of family members who have had lice infestation and itching is reported in infested girls more than uninfested girls with a significant difference (*p* < 0.05) (Table 1).

### 3.2. The Efficacy of Antilice Shampoos Against Lice Infestation in Infested Girls

From 77 infested girls, 72 girls (93.5%) obtained permission from guardians to take part in this study. The efficacy of antilice shampoos showed a 5.6% cure rate after one-time treatment. Three-time treatment showed superior results of a 73% cure rate.

Chemical antilice shampoo with permethrin ingredient showed similar results of cure rate as herbal antilice shampoo with not significantly different statistical value (*p* > 0.05), as shown in Table 2.

### 3.3. The Side Effects and Satisfaction Regarding Antilice Shampoo

There was no side effect of shampoo treatment (95.8%), and 4.2% reported no serious sign of side effects from both antilice shampoos, 2.8% of itching, and 1.4% of a burning sensation (Table 3).

More than half of the participants showed undecided satisfaction. Just 2.8% disliked antilice shampoo, and the most disliked was herbal shampoo (Table 4).

### 3.4. Reinfestation of Head-Lice After Treatment for 2 and 12 Months

Forty-four treated girls were able to be followed up in 2 months. Eight girls out of 44 showed repeated infestation with head lice (18.2%). After 12 months of treatment, 12 out of 35 (34.3%) girls showed lice reinfestation.

## 4. Discussion

Since 2017, in a suburban area of Pathum Thani, pediculosis still continues showing a high prevalence (68.7–89.2%) [12]. Even though a campaign to provide a single treatment with antilice shampoo (0.5% permethrin w/w) with fine combs was given to infested girls for several years, lice infestation was considered to be a serious problem in suburban communities, where the parents/guardians (~50%) had no time to look after the kids. Our study showed a high prevalence of 50.3% of pediculosis in schoolgirls at Levels 3–6 in a suburban school in Pathum Thani, which was lower than another school (87.5%) in Lak Hok, Pathum Thani [12]. The outbreak prevalence of head lice was higher than previously reported elsewhere (15.1%–47.1%) in Thailand, such as in some areas of Bangkok [6], Northern area [13], Ubon Ratchathani Province [34], and in rural areas of Thailand [11].

A high prevalence in an urban school in this area cannot be overlooked. There is a need to combine the strategy to eliminate lice infestation, supported by teachers, students, parents, and the health ministry. One way to eliminate and control lice infestation is by treatment using safe insecticides [5]. The most common insecticide group is pyrethroids, sold under the trade name permethrin [35, 36]. However, recently, due to incomplete treatment, lice have become resistant [37–39]. On the other hand, the standard lotion takes a long time for application (24 h) to treat head lice, leading to treatment failure that might be attributed to lice resistance to insecticides [39].

This study reveals the efficacy of two antilice shampoos, reaching a 73% cure rate in infested girls when used consecutively 3 times (1 time a week). One-time use shampoo is unable to kill all forms of lice (5.6% of cure rate). Similar to Tanthanapanyakorn et al. [29], a longer treatment time is needed to get better efficacy of pediculosis elimination. Despite this, we also observed 4 times reapplication of shampoos did not increase the cure rate of infested cases, similar to another study that applied the antilice herbal for 2–3 days and showed no difference in the efficacy of treatment [29]. Treatment must be repeated after 7 days to kill newly hatched nymphs from eggs [40].

Herbal antilice shampoos showed a similar cure rate to chemical antilice shampoos containing permethrin (*p* > 0.05). Various studies show high efficacy of antilice herbs against head lice [25–27]. These might be an alternative choice, using antilice herbal instead of chemical shampoos, since they are available locally at low cost, easy to use, have a short exposure time of between 5 and 10 mins, and demonstrate no major side effects apart from a few cases of itching and burning sensation.

It was noted that some girls complained of dry hair after application, and shampoo is too liquid and does not stick to the hair which makes it hard to use, causing half of the participants to show undecided satisfaction. This needs to be considered when buying antilice shampoos.

Lice reinfestation was 18.2% after 2 months and increased to 34.3% after 12 months. According to informal interviews, reinfestation of lice in girls may be caused by improper hair washing and infestation by family members and friends. Support from the family and the school may be necessary to prevent reinfestation. Unpublished data in Thailand (2014) revealed lice reinfestation was 46% after 2 months, and it reduced to 21% when combining lice treatment with intervention and activities in a school. Also, in Brazilian patients, it was reported that repeated infestations can occur: 15–19 infestations annually [41]. This might be promising for surveillance strategies. Furthermore, the treatment might be introduced to the whole family of infested cases, and the knowledge of lice should be taught in school lessons and activities. If everyone keeps the lessons in mind and is involved in the activities, this might be the way to prevent the recurrence of head lice.

## 5. Conclusion

A high prevalence of lice infestation (50.3%) was reported in primary school girls (Levels 3–6) in Pathum Thani Province in Thailand. Herbal antilice shampoo showed a high efficacy of treatment equal to chemical shampoo. Three times reapplications showed a high efficacy to eliminate lice more than 1–2 times. Reinfestation ranged from 18.2% to 34.3% in 2 and 12 months after treatment, respectively. This finding might send a message to schools to find proper strategies to prevent and eliminate the head lice infestation.

## Figures and Tables

**Table 1:** General characteristics of primary school students at Levels 3–6 and the prevalence of head lice infestation in a high prevalence school, Pathum Thani Province.

**Characteristics**	**Lice infestation (%)** **n** = 78** (21.9)**	**No infestation (%)** **n** = 278** (78.1)**	**Total (%)** **n** = 356** (100.0)**	**χ**2** -value**	**p** ** value**
Gender					
Boy	1 (0.5)	202 (99.5)	203 (57.0)	126.635	0.001
Girl	77 (50.3)	76 (49.7)	153 (43.0)		
Primary school level					
3	18 (17.8)	83 (82.2)	101 (28.4)	2.374	0.499
4	24 (25.5)	70 (74.5)	94 (26.4)		
5	22 (24.7)	67 (75.3)	89 (25.0)		
6	14 (19.4)	58 (80.6)	72 (20.2)		
History of lice infestation					
No	6 (2.9)	199 (97.1)	205 (57.6)	101.796	0.001
Yes	72 (28.7)	79 (52.3)	151 (42.4)		
Lice infestation in family					
No	47 (17.7)	219 (82.3)	266 (74.7)	10.100	0.002
Yes	31 (34.4)	59 (65.6)	90 (25.3)		
Itching sign					
No	15 (6.3)	224 (93.7)	239 (67.1)	103.887	0.001
Yes	63 (53.8)	54 (46.2)	117 (32.9)		

**Table 2:** The efficacy of two antilice shampoos against lice infestation (three times treatment).

**Antilice shampoos**	**No. of negative cases after treatment/total no. of infested girl test (%)**		
	**Day 7** **n** = 72	**Day 14** **n** = 70	**Day 21** **n** = 63
Chemical shampoo with permethrin	3/36 (8.3)	13/35 (37.1)	24/31 (77.4)
Herbal shampoo	1/36 (2.8)	11/35 (31.4)	22/32 (68.8)
Total	4/72 (5.6)	24/70 (34.3)	46/63 (73.0)
*χ*2-value	1.059	0.254	0.601
*p* value	0.589	0.881	0.438

**Table 3:** Side effect of using antilice shampoo (*n* = 72).

**Antilice shampoos**	**No. of infested girls with side effects (%)**		
	**No side effect**	**Itching**	**Burning sensation**
Chemical shampoo with permethrin (*n* = 36)	35 (97.2)	1 (2.8)	0 (0)
Herbal shampoo (*n* = 36)	34 (94.4)	1 (2.8)	1 (2.8)
Total (*n* = 72)	69 (95.8)	2 (2.8)	1 (1.4)

**Table 4:** Satisfaction of antilice shampoo in infested girls (*n* = 72).

**Antilice shampoos**	**No. of cases answer (%)**			
	**Like**	**Undecided**	**Dislike**	**No answer**
Chemical shampoo with permethrin (*n* = 36)	3 (8.3)	29 (80.6)	0 (0.0)	5 (13.9)
Herbal shampoo (*n* = 36)	5 (13.9)	18 (50.0)	2 (5.6)	11 (30.6)
Total (*n* = 72)	8 (11.1)	47 (65.3)	2 (2.8)	16 (22.2)

## Data Availability

The data used to support the findings of this study are included within the article.

## References

[1] Gratz N. G. (1997). *Human Lice, Their Prevalence, Control and Resistance to Insecticides*.

[2] Ko C. J., Elston D. M. (2004). Pediculosis. *Journal of the American Academy of Dermatology*.

[3] Falagas M. E., Matthaiou D. K., Rafailidis P. I., Panos G., Pappas G. (2008). Worldwide Prevalence of Head Lice. *Emerging Infectious Diseases*.

[4] Hatam-Nahavandi K., Ahmadpour E., Pashazadeh F. (2020). Pediculosis Capitis Among School-Age Students Worldwide as an Emerging Public Health Concern: A Systematic Review and Meta-Analysis of Past Five Decades. *Parasitology Research*.

[5] Coates S. J., Thomas C., Chosidow O., Engelman D., Chang A. Y. (2020). Ectoparasites: Pediculosis and Tungiasis. *Journal of the American Academy of Dermatology*.

[6] Rassami W., Soonwera M. (2012). Epidemiology of Pediculosis Capitis Among Schoolchildren in the Eastern Area of Bangkok, Thailand. *Asian Pacific Journal of Tropical Biomedicine*.

[7] Leung A. K., Fong J. H., Pinto-Rojas A. (2005). Pediculosis Capitis. *Journal of Pediatric Health Care*.

[8] Willems S., Lapeere H., Haedens N., Pasteels I., Naeyaert J.-M., De Maeseneer J. (2005). The Importance of Socio-Economic Status and Individual Characteristics on the Prevalence of Head Lice in SchoolChildren. *European Journal of Dermatology*.

[9] Burkhart C. G., Burkhart C. N., Burkhart K. M. (1998). An Assessment of Topical and Oral Prescription and Over-the-Counter Treatments for Head Lice. *Journal of the American Academy of Dermatology*.

[10] AlBashtawy M., Hasna F. (2012). Pediculosis Capitis Among Primary-School Children in Mafraq Governorate, Jordan. *Eastern Mediterranean Health Journal*.

[11] Singhasivanon O. U., Lawpoolsri S., Mungthin M., Yimsamran S., Soonthornworasiri N., Krudsood S. (2019). Prevalence and Alternative Treatment of Head-Lice Infestation in Rural Thailand: A Community-Based Study. *Korean Journal of Parasitology*.

[12] Kitvatanachai S., Kritsiriwutthinan K., Taylor A., Rhongbutsri P. (2023). Head Lice Infestation in Pre-High School Girls, Lak Hok Suburban Area, Pathum Thani Province, in Central Thailand. *Journal of Parasitology Research*.

[13] Ruankham W., Winyangkul P., Bunchub N. (2016). Prevalence and Factors of Head Lice Infestation Among Primary School Students in Northern Thailand. *Asian Pacific Journal of Tropical Disease*.

[14] Thanyavanich N., Maneekan P., Yimsamram S., Maneeboonyang W., Puangsa-art S., Wuthisen P. (2009). Epidemiology and Risk Factors of Pediculosis Capitis in Primary Schools Near the Thai-Myanmar Border in Ratchaburi Province, Thailand. *Journal of Tropical Medicine and Parasitology*.

[15] Tebruegge M., Pantazidou A., Curtis N. (2011). What’s Bugging You? An Update on the Treatment of Head Lice Infestation. *Archives of Disease in Childhood-Education and Practice*.

[16] Sangare A. K., Doumbo O. K., Raoult D. (2016). Management and Treatment of Human Lice. *BioMed Research International*.

[17] Walker C., Sebastian R., Krishna S., Tobias J. D. (2016). A Lousy Reason for Surgery Cancellations. *Clinical Pediatrics*.

[18] Brownell N., Sunantaraporn S., Phadungsaksawasdi K. (2020). Presence of the Knockdown Resistance (*kdr*) Mutations in the Head Lice (*Pediculus humanus capitis*) Collected From Primary School Children of Thailand. *PLoS Neglected Tropical Diseases*.

[19] Food and Drug Administration (2017). “Drug Information” [in Thai]. https://bit.ly/2RlyBZU.

[20] Feldmeier H. (2012). Pediculosis Capitis: New Insights Into Epidemiology, Diagnosis and Treatment. *European Journal of Clinical Microbiology & Infectious Diseases*.

[21] Ahmad H. M., Abdel-Azim E. S., Abdel-Aziz R. T. (2014). Assessment of topical versus oral ivermectin as a treatment for head lice. *Dermatologic Therapy*.

[22] Downs A. M. (2004). Managing Head Lice in an Era of Increasing Resistance to Insecticides. *American Journal of Clinical Dermatology*.

[23] Amanzougaghene N., Fenollar F., Nappez C. (2018). Complexin in Ivermectin Resistance in Body Lice. *PLoS Genetics*.

[24] Larkin K., Rodriguez C. A., Jamani S. (2020). First Evidence of the Mutations Associated With Pyrethroid Resistance in Head Lice (Phthiraptera: Pediculidae) From Honduras. *Parasites & Vectors*.

[25] Soonwera M. (2014). Efficacy of Herbal Shampoo Base on Native Plant Against Head Lice (*Pediculus humanus capitis* De Geer, Pediculidae: Phthiraptera) In Vitro and In Vivo in Thailand. *Parasitology Research*.

[26] Intaranongpai J., Warinthorn C., Wandee G. (2006). Anti-Head Lice Effect of Annona Squamosa Seeds. *The Southeast Asian Journal of Tropical Medicine and Public Health*.

[27] Inthaphalan K., Raksamat W., Kamoltham T. (2022). Efficacy and Safety of Indian Mulberry Leaf Mixed With Galanga Extract Shampoo Versus Benzyl Benzoate Lotion for the Treatment of Head Lice Infestation. *Thai Pharmaceutical and Health Science Journal*.

[28] Yingklang M., Gordon C. N., Jaidee P. H., Thongpon P., Pinlaor S. (2023). Comparative Efficacy of Chemical and Botanical Pediculicides in Thailand and 4% Dimeticone Against Head Louse, *Pediculus humanus capitis*. *PLoS One*.

[29] Tanthanapanyakorn P., Pathomrotsakun J., Jumneansuk A., Jaricksakulchai J., Butratsamee A., Meechumnan R. (2019). Efficacy of Herbal Extracts for Eliminating Pediculosis Capitis in Primary School Children, Wat Mong Khon Rat School, Pathum Thani Province. *Journal of Nursing and Education*.

[30] Roberts R. J. (2002). Head Lice. *New England Journal of Medicine*.

[31] Soleimani-Ahmadi M., Jaberhashemi S. A., Zare M., Sanei-Dehkordi A. (2017). Prevalence of Head Lice Infestation and Pediculicidal Effect of Permethrine Shampoo in Primary School Girls in a Low-Income Area in Southeast of Iran. *BMC Dermatology*.

[32] El-Bahnasawy M. M., Abdel F. E., Morsy T. A. (2013). Human Pediculosis: A Critical Health Problem and What About Nursing Policy?. *Journal of the Egyptian Society of Parasitology*.

[33] CDC (2023). Head lice: Diagnosis. https://www.cdc.gov/parasites/lice/head/diagnosis.html.

[34] Anusai N., Somprasong N., Hanjangsit K., Maleelai K. (2016). Epidemiology of Pediculosis Capitis in Primary School Students in Warinchumrab District, Ubon Ratchathani Province. *Proceedings of the 12th Mahasarakham University Research Conference; 2016*.

[35] Burgess I. F., Brunton E. R., Burgess N. A. (2013). Single Application of 4% Dimeticone Liquid Gel Versus Two Applications of 1% Permethrin Creme Rinse for Treatment of Head Louse Infestation: A Randomised Controlled Trial. *BMC Dermatology*.

[36] Feldmeier H. (2014). Treatment of Pediculosis Capitis: A Critical Appraisal of the Current Literature. *American Journal of Clinical Dermatology*.

[37] Dong K., du Y., Rinkevich F. (2014). Molecular Biology of Insect Sodium Channels and Pyrethroid Resistance. *Molecular Biology*.

[38] Eremeeva M. E., Capps D., Winful E. B. (2017). Molecular Markers of Pesticide Resistance and Pathogens in Human Head Lice (Phthiraptera: Pediculidae) From Rural Georgia, USA. *Journal of Medical Entomology*.

[39] Abbasi E., Daliri S., Yazdani Z. (2023). Evaluation of Resistance of Human Head Lice to Pyrethroid Insecticides: A Meta-Analysis Study. *Heliyon*.

[40] Meister L., Ochsendorf F. (2016). Head Lice. *Deutsches Ärzteblatt International*.

[41] Bohl B., Evetts J., McClain K., Rosenauer A., Stellitano E. (2015). Clinical Practice Update: Pediculosis Capitis. *Journal of Pediatric Nursing*.

